# Target-Sequencing of Female Infertility Pathogenic Gene Panel and a Novel TUBB8 Loss-of-Function Mutation

**DOI:** 10.3389/fgene.2022.865103

**Published:** 2022-05-10

**Authors:** Hongxia Yuan, Jianhua Chen, Na Li, Hui Miao, Yao Chen, Shuyan Lyu, Yu Qiao, Guangping Yang, Hui Luo, Liangliang Chen, Fei Mao, Lingli Huang, Yanni He, Saifei Hu, Congxiu Miao, Yun Qian, Ruizhi Feng

**Affiliations:** ^1^ State Key Laboratory of Reproductive Medicine, Nanjing Medical University, Nanjing, China; ^2^ The Reproduction Engineer Key Laboratory of Shanxi Health Committee, Department of Reproductive Genetics, Institute of Reproduction and Genetics of Changzhi Medical College, Heping Hospital of Changzhi Medical College, Changzhi, China; ^3^ Reproductive Medical Center of the Second Affiliated Hospital of Nanjing Medical University, Nanjing, China; ^4^ The Affiliated Huaian No. 1 People’s Hospital of Nanjing Medical University, Huaian, China

**Keywords:** genetic screening, female infertility, target sequencing, TUBB8, mutation

## Abstract

Genetic screening is an important approach for etiology determination and helps to optimize administration protocols in reproductive centers. After the first pathogenic gene of female infertility was reported in 2016, more and more new pathogenic genes were discovered, and we sought to develop an efficient and cost-effective method for genetic screening in patients. In this study, we designed a target-sequencing panel with 22 female infertility-related genes, namely, TUBB8, PATL2, WEE2, and PANX1 and sequenced 68 primary infertility (PI) and recurrent pregnancy loss (RPL) patients. We sequenced 68 samples reaching an average depth of 1559× and detected 3,134 variants. Among them, 62.2% were synonymous single-nucleotide variants (SNVs) and 36.3% were non-synonymous SNVs. The remaining 1.5% are indels (insertions and deletions) and stop-gains. DNAH11 and TUBB8 are the two genes that mutated most frequently. We also found a novel TUBB8 variant (c.898_900del; p.300_300del), proved its loss-of-function mechanism, and profiled the interactome of the wild-type (WT) and mutant TUBB8 proteins. Overall, this target-sequencing method provides an efficient and cost-effective approach for screening in IVF clinics and will support researchers for the discovery of new pathogenic variants.

## Introduction

Genetic screening is a newly developed technology based on the widespread next-generation sequencing (Pös et al., 2019). Compared with the comprehensive but expensive approach of genome sequencing, exome sequencing has already been used for new pathogenic genes discovery in patient cohorts and preimplantation genetic testing in reproductive centers. For known genetic disorders, the simple and cost-effective method to detect pathogenic genes is target sequencing. Designing hybridization probes or multiple PCR primers, genes of interest can be enriched and sequenced, thus enabling researchers to detect variants in hot-spot regions. Targeted sequencing has been widely used in numerous areas such as genetic disorders (Eggers et al., 2016), pharmacogenetics (Gordon et al., 2016), and cancer (Matsunaga, 2009). With profiles of actionable gene alterations, the ovarian cancer target sequencing makes it possible to find variants responding to molecular targeting drugs. Tumors containing variants on PIK3CA, AKT1, and PTEN are targetable by P13K/AKT/mTOR inhibitors (Takenaka et al., 2015). Another crucial application is prenatal screening. While traditional ultrasonography at the latter stage of pregnancy is usually used to diagnose skeletal dysplasia, target sequencing of recurrent pathogenic variants in FGFR3, COL1A2, etc. in the early gestational stage meets the need of precise diagnosis of skeletal dysplasia (Ching-Yuan Wang et al., 2021).

Infertility affects about 48.5 million couples in the world (Chiware et al., 2021). Among multiple factors contributing to this complex disease, genetic causes have been drawn increasing attentions (Miyamoto et al., 2017; Beke et al., 2019). Genetic screening has been applied in some reproductive centers for years, especially in male infertile patients (Liu et al., 2020). Screening in genetic abnormalities, such as Y microdeletions and chromosomal aberration, and known pathogenic genes (CFTR for obstructive azoospermia, NR5A1 in disorders of sex development, etc.), have been routinely performed for decades (An et al., 2021). However, the pathogenic genes of female infertility were hidden until the first gene TUBB8 was reported to cause oocyte maturation arrest (Feng et al., 2016). Since then, a number of genes have been discovered in primary infertility (PI) and recurrent pregnancy loss (RPL) patients, namely, PATL2 (Chen et al., 2017a), TRIP13 (Zhang et al., 2020a), WEE2 (Sang et al., 2018), etc. These newly found genes contribute to female infertile phenotypes including oocyte maturation arrest, zygotic cleavage failure, and embryo developmental arrest. Screening of these genes in patients with idiopathic assisted reproduction failures would greatly help in diagnosis and genetic counseling.

In this study, we designed a target sequencing gene panel for detecting pathogenic genes of female infertility. A total of 68 PI/RPL patients were recruited, sequenced, and analyzed. We found two TUBB8 variants, namely, a novel single-nucleotide variant (SNV), and demonstrated its loss-of-function pathogenic mechanism. Interactome of wild-type (WT) and mutant TUBB8 proteins were also profiled. This female infertility gene panel would be an efficient and cost-effective tool for genetic screening and diagnosis, as well as a method for researchers to discover more variants, and further study genetic pathogenicity of female infertility.

## Materials and Methods

### Human Subjects, Standards of Inclusion and Exclusion, and Ethical Approval

Patients were recruited from the Clinical Center of Reproductive Medicine, the second affiliated hospital of Nanjing Medical University, the affiliated Huaian No. 1 People’s Hospital of Nanjing Medical University, and Heping Hospital affiliated to Changzhi Medical College during 2020.1–2021.10. All the recruited patients had a history of PI or RPL. The age of the participated patients was no more than 38 years. They were not diagnosed with other complex reproductive diseases such as polycystic ovarian syndrome, premature ovarian failure, and premature ovarian insuffificiency. No chromosome defects or infertile factors were found in their spouses. Peripheral blood (5 ml) was collected from each patient. All participating patients gave informed consent. This study was approved by the Ethics Committee of Nanjing Medical University.

### Panel Characterization and Information of Each Gene

**FIGURE 1:**
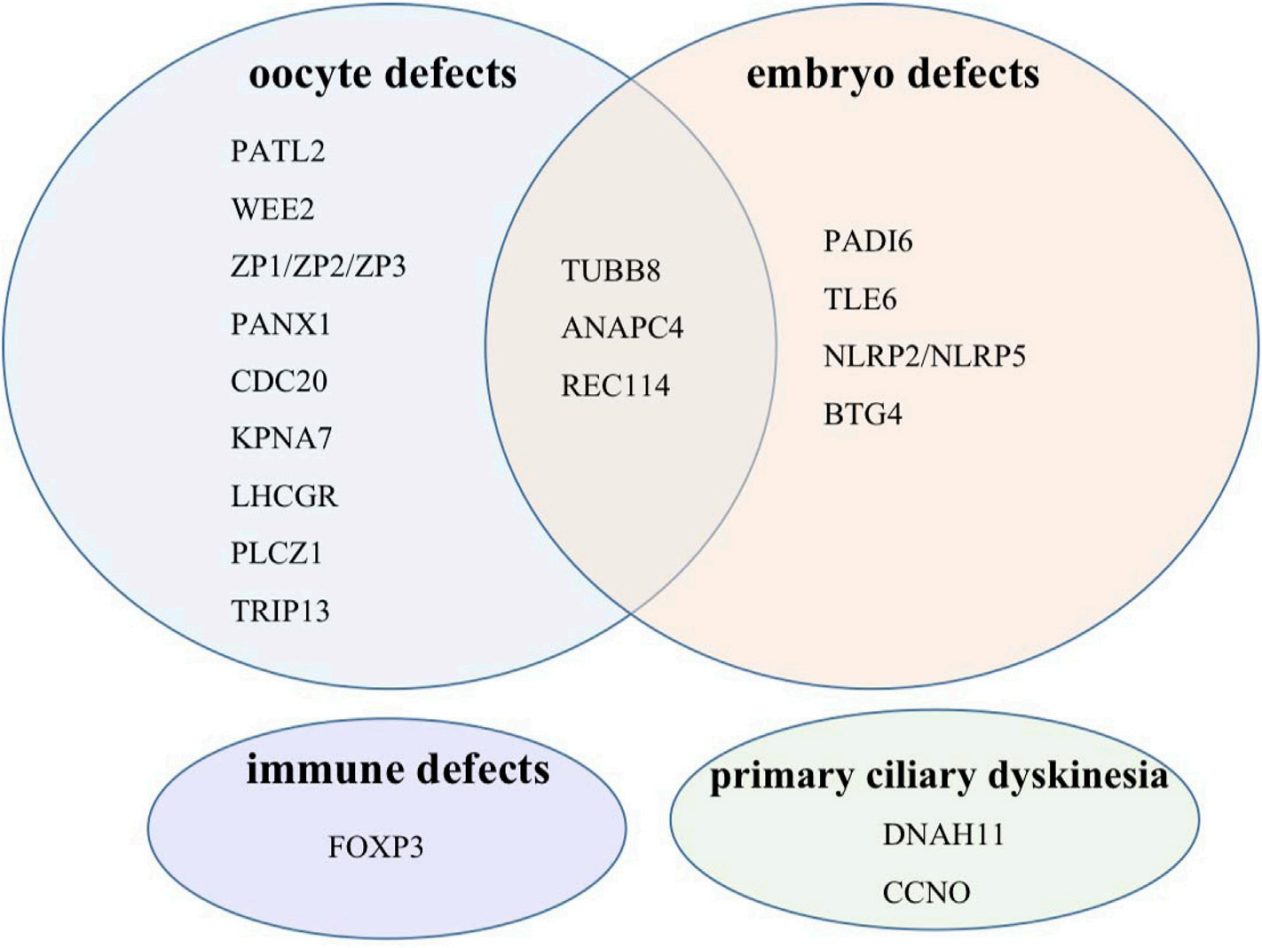
Target genes classified into four categories according to their phenotypes.

Our gene panel includes 22 female infertility-related genes: TUBB8, PATL2, WEE2, PADI6, TLE6, ZP1, ZP2, ZP3, NLRP2, NLRP5, PANX1, REC114, PLCZ1, CDC20, ANAPC4, TRIP13, KPNA7, BTG4, DNAH11, CCNO, LHCGR, and FOXP3 (Sang et al., 2021; Mu et al., 2020; Furuta et al., 2000; Wang et al., 2012; Zhang et al., 2020b; Weijie Wang et al., 2021). Detailed information of each gene is shown in Table 1. According to their phenotypes, these genes are classified into four categories (Figure 1). Some genes such as TUBB8 have spectrums of phenotypes, which means that they could be responsible for more than one female infertility phenotype (Chen et al., 2017b).

**TABLE 1:** Genes included in the target sequencing panel and their phenotypes.

Gene	Phenotype	Locus	OMIM	30× coverage (%)
TUBB8	Oocyte maturation defects	10p15.3	616768	100
PATL2	Oocyte maturation defects	15q21.1	614661	100
WEE2	Fertilization failure	7q34	614084	100
PADI6	Early embryo arrest	1p36.13	610363	100
TLE6	Early embryo arrest	19p13.3	612399	100
ZP1	Abnormal zona pellucida formation	19p13.3	195000	100
ZP2	Abnormal zona pellucida formation	16p12.3-p12.2	182888	100
ZP3	Abnormal zona pellucida formation	7q11.23	182889	100
NLRP2	Early embryo arrest	19q13.42	609364	100
NLRP5	Early embryo arrest	19q13.43	609658	100
PANX1	Oocyte death	11q21	608420	100
REC114	Oocyte death	15q24.1	618421	100
PLCZ1	Fertilization failure	12p12.3	608075	100
CDC20	Meiosis defects	1p34.2	603618	100
ANAPC4	Abnormal gametogenesis and embryogenesis	4p15.2	606947	100
TRIP13	Oocyte maturation defects	5p15.33	604507	100
KPNA7	Fertilization failure	7q22.1	614107	100
BTG4	Zygotic cleavage failure	11q23.1	605673	100
LHCGR	Abnormal zona pellucida formation	2p16.3	152790	100
FOXP3	Immunodysregulation, polyendocrinopathy	Xp11.23	300292	100
DNAH11	Primary ciliary dyskinesia	7p15.3	603339	100
CCNO	Primary ciliary dyskinesia	5q11.2	607752	100

### Genomic DNA Extraction and Target-Panel Sequencing

Genomic DNA was extracted from the peripheral blood using TIANamp Genomic DNA KIT (TIANGEN, DP304-03) following the manufacturer’s instructions. DNA concentrations were detected on Nanodrop 2000. Concentrations above 25 µg/µl and OD_260/280_ between 1.8 and 2.0 were used for the next steps. DNA qualities were tested with agarose gel electrophoresis. Specialized DNA probes were designed according to the coding regions of the target genes. Hybridization capture and enrichment were performed to screen candidate variants (iGeneTech Ltd. Co., China). Sequencing was performed on Illumina NovaSeq 6000 platform (iGeneTech Ltd. Co., China). Raw reads were filtered to remove low-quality reads. Q30 ≥80% was regarded as qualified. Clean data were mapped using BWA (Burrows-Wheeler Alignment tool), and statistical analyses were conducted (iGeneTech Ltd.Co., China). Candidate variants were selected for further analysis based on the following: variants located in exons or splicing regions, non-synonymous, and non-benign as predicted by InterVar. The left variants were further screened with minor allele frequency no more than 0.1% in at least one database such as NHLBI-ESP, 1000G, ExAC, gnomAD, and the in-house database of iGeneTech. Sanger sequencing was performed for validation.

### Plasmid Construction, Cell Culture, and Transfection

Eukaryotic expression vectors pCMV6 with Myc-DDK (FLAG) tags containing WT and mutant TUBB8 were kindly provided by Professors Lei Wang and Qing Sang in Fudan University. We recombined the vector to add a C-terminal AviTag for protein purification. pEF1a-BirA-V5-neo was purchased from MiaoLing Plasmid Platform. Variants were introduced using Mut Express II Fast Mutagenesis Kit 2 (Vazyme, C214). The HEK293T cell line was purchased from National Collection of Authenticated Cell Culture and cultured with DMEM adding 10% fetal bovine serum and penicillin/streptomycin at 37°C with 5% CO_2_. The cells were transiently transfected using Lipofectamine 2000 reagent following the standard protocols.

### Immunofluorescence and Confocal Microscopy

Twenty-four hours after seeding, HEK 293T cells were transiently transfected with WT and mutant C-terminally Myc-DDK (FLAG)–tagged TUBB8 plasmids. The cells were gently washed three times using PBS 48 h after transfection. Then, 2% paraformaldehyde was applied to fix the cells. Permeabilization was performed with 0.5% Triton X-100 for 30 min at room temperature after being washed with PBS three times. Nonspecific binding was blocked by 5% BSA at room temperature for 1 h. The cells were then incubated in the dark at 4°C overnight with diluted antibodies: anti-FLAG M2-Cy3 (sigma, #A9594, 1:500), anti-α tubulin (cell signaling technology, #5063S, Alexa flour 488 conjugated, 1:250), and DAPI (Beyotime, #C1002, 1:1000). The cells were washed three times using 0.5% Triton X-100, adding 0.5% Tween-20 before mounting. The confocal images were taken using Leica TCS SP8 confocal laser scanning microscope.

### Cells Lysis and Western Blots

Buffers used for cell harvest and purification were in reference to *Cytoskeleton Dynamics* (Maiato, 2020). Forty-eight hours post-transfection, cells were gently washed once by cold D-PBS (Beyotime, #C0221D), and then 3 ml fresh D-PBS was added to collect the cells into a new 15-ml conical centrifuge tube by pipetting up and down. Centrifugation at 200 *g* for 10 min was performed, and cold lysis buffer was added after entirely removing D-PBS. At 4°C, cells lysate was rotated for 30 min and centrifuged at 14000 *g* for 10 min. The supernatant was saved to measure concentrations with BCA protein assay kit (Beyotime, #P0012). 10% SDS-PAGE gel was applied, and proteins were then transferred to the PVDF membranes. Nonspecific binding was blocked by 5% BSA (Beyotime, #ST023) at room temperature for 1 h before incubating in diluted primary antibodies overnight at 4°C: anti-Vinculin (Abcam, #ab129002, 1:10000), anti-FLAG tag mouse monoclonal antibody (YIFEIXUE BIOTECHNOLOGY, #YFMA0036, 1:5000), anti-Myc tag antibody (Abcam, #ab32, 1:1000), TBCD polyclonal antibody (Proteintech, #14867-1-AP, 1:2000), TBCA polyclonal antibody (Proteintech, #12304-1-AP, 1:2000), and HRP-Streptavidin (Beyotime, #A0303, 1:100000). Secondary antibodies, HRP-conjugated goat anti-mouse IgG (YIFEIXUE BIOTECHNOLOGY, #YFSA01, 1:7500) or HRP-conjugated mouse anti-rabbit IgG (Sangon Biotech, #D110065, 1:7500), were used to incubate the membranes for 1 h at room temperature, and then washed three times in Tris-buffered saline containing 0.05% Tween-20. Chemistar^™^ High-sig ECL Western Blotting Substrate (Tanon, #180-5001) was used to detected immune complexes on Tanon 4500 SF.

### Protein Purification, Silver Staining, and Mass Spectrometry

Cells in 10-cm dishes were co-transfected equimolar amounts of C-terminal AviTag WT/mutant TUBB8 pCMV6 vectors and pEF1a-BirA-V5-neo. The protocols for cell harvest and lysis were the same as WB. After determining the protein concentration, equal amounts of protein were purified by Dynabeads^™^ M280 Streptavidin (Invitrogen, #11205D), according to the protocols from the *Cytoskeleton Dynamics* (Maiato, 2020). Approximately, 30 μl of beads were treated with blocking buffer for 1 h at room temperature. Six milligrams of proteins was added into the beads and rotated at 4°C for 2 h. Subsequently, the beads were washed seven times with a wash buffer before being boiled with 40 µl of SDS-PAGE sample-loading buffer (Beyotime, #P0015A) at 95°C for 5 min. 10% SDS-PAGE was exploited to separate purified proteins. Silver staining was performed with a commercial kit following the standard instructions (Beyotime, #P0017S). Protein analysis was accomplished with shotgun mass spectrometry by Shanghai Bioprofile.

### Statistical Analysis

GO enrichment analysis was performed and visualized by clusterProfiler R package (4.0) (Wu et al., 2021).

## Results

### Gene Panel Performance Evaluation and Sequencing Data Analysis

To evaluate the performance of our panel capture and sequencing, the average values of relevant parameters were reported as below. The QC rate [clean bases (Mb)/raw bases (Mb)] was 91.07%, and the total reads mapping rate (mapped reads/clean reads) was 98.9%. The target reads capture rate (target reads/mapped reads) was 57.1%. The target effective rate [target effective bases (Mb)/total effective bases (Mb)] was 34.15%. The mean depth of panel sequencing is 1559×, and the 30× coverage is 100%. T 10% ×coverage, which meant about 155× coverage rate was 99.97%, and T 50% ×coverage was 92.56% (Supplementary Table S1). Qualified data by target sequencing laid a solid foundation for the precision of subsequent analysis.

**FIGURE 2:**
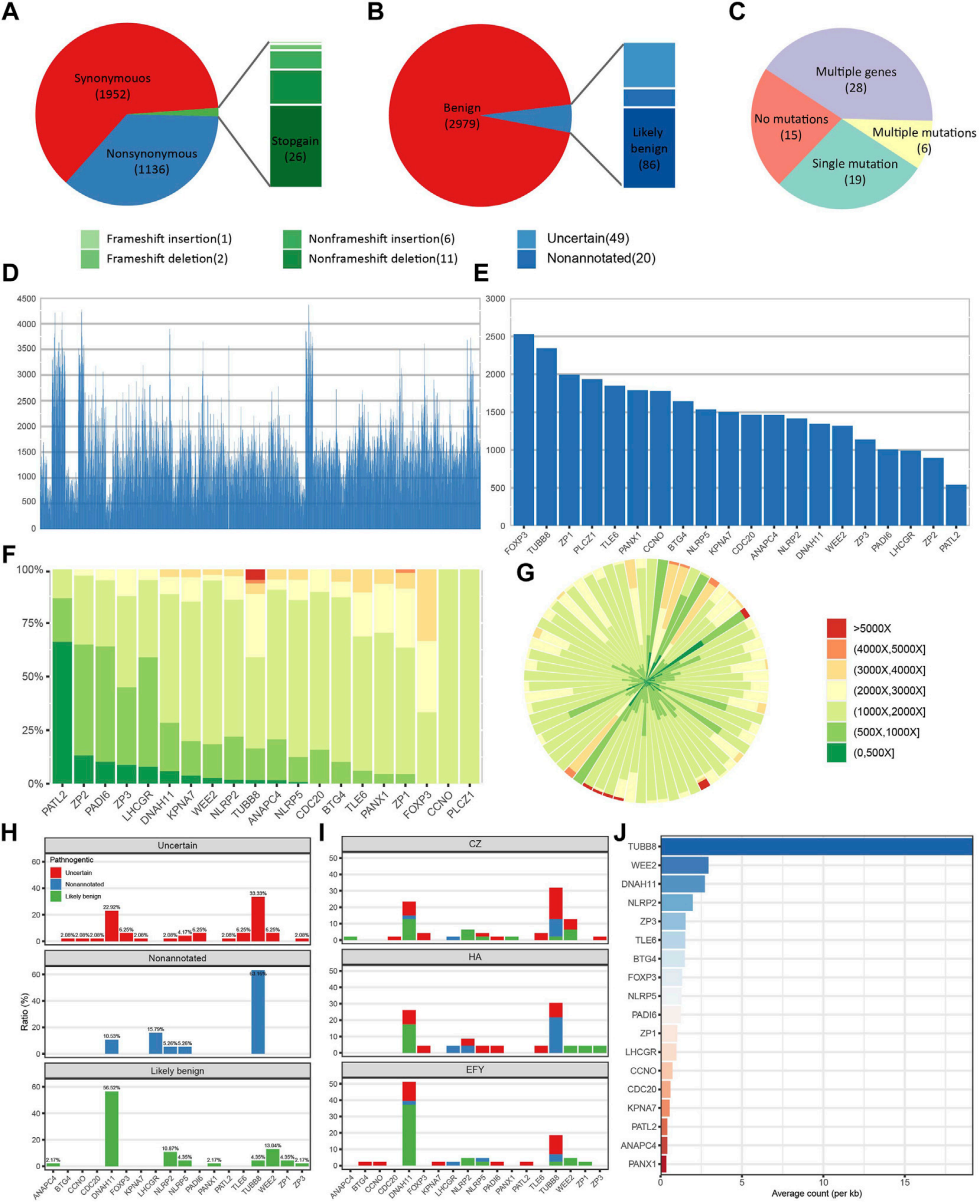
Panel sequencing performance. **(A)** The proportions of different types of variants. **(B)** The proportions of variants with different annotations. **(C)** The distribution of patients with different amounts of potentially significant variants. **(D)** Sequencing depth of all detected exonic and splicing variants. **(E)** Average sequencing depth of each gene. **(F)** Sequencing depth distribution of variants on each gene. **(G)** Sequencing depth distribution of each patient. **(H)** Annotation distributions of all non-synonymous and non-benign variants on each gene. **(I)** Distributions of all non-synonymous and non-benign variants on each gene from three different reproductive centers. CZ means Heping Hospital affiliated to Changzhi Medical College. HA means the affiliated Huaian No. 1 People’s Hospital of Nanjing Medical University, and EFY means Reproductive Medical Center of the Second Affiliated Hospital of Nanjing Medical University. **(J)** Normalized variants distribution on each gene.

An average of 14.2 variant-bearing genes were detected per patient. A total of 3,134 variants located in exons and splicing regions were found, in which 3,088 were SNVs and 46 were indels. In 3,088 SNVs, 1952 were synonymous and 1136 variants were non-synonymous. In 46 indels, there were 26 stop-gain, 11 non-frameshift deletions, 2 frameshift deletions, 6 non-frameshift insertions, and 1 frameshift insertion (Figure 2A). Based on clinical interpretation, 2,979 out of 3,134 were benign according to the ACMG (the American College of Medical Genetics and Genomics) and InterVar, while 86 variants were likely benign and 49 had uncertain significance, leaving 20 of them non-annotated (Figure 2B). We excluded all the synonymous and benign variants, and we focused on those non-synonymous and non-benign variants which we referred to as “potentially significant” variants. In 68 patients, 15 patients had no potentially significant variants, and 19 patients had only a single potentially significant variant. Moreover, 6 patients had multiple variants on a single gene, while 28 patients had multiple variants on multiple genes (Figure 2C). The sequencing depth of all splicing and exonic variants showed that 74.0% of them had reached the depth of over 1000× (Figure 2D). The gene with the highest average depth was FOXP3, which was above 2500×. PATL2 had the lowest average depth, which was around 500× (Figure 2E). REC114 and TRIP13 had no detected exonic and splicing variants. Variants sequencing depth distribution displayed that most variants were sequenced between 1000× and 2000×. PATL2 had about 60% variants sequenced no more than 500×, while some variants from TUBB8 were sequenced deeper than 5000× (Figure 2F). Figure 2G showing the depth distribution of each patient demonstrated that most sequencing depth was between 1000× and 2000×.

Based on clinical annotation, TUBB8 had the highest percentage of non-annotated and uncertain variants, and DNAH11 was the highest in the likely benign variants (Figure 2H). Data from three different reproductive centers showed that TUBB8 and DNAH11 were the top two most frequently variant genes (Figure 2I). Mutant frequencies of each gene were changed when we normalized the counts of variants in gene length. However, TUBB8 was still the most frequently variant gene (Figure 2J). Our target gene panel sequencing exhibited good quality in sequencing depth and coverage, though optimization is in need for better performance.

### Sanger Sequencing Validation

Among the potentially significant variants we detected, when FRE (the proportion of variants bases in all detected bases) was above 0.4, the results of panel sequencing were 100% validated. We noticed that variants with FRE around 0.2 could not be validated (Supplementary Figure S1). This is probably due to the sensitivity limitation of Sanger sequencing. When FRE was low, PCR amplification bias made Sanger sequencing hard to detect low-frequency variants. We also found another frequent variant TUBB8 (c.167-169del:p.56-57del), which was reported in nine patients and could not be validated. It may be because this region captured by the probes is highly similar to other β-tubulin isotype genes or some duplicated fragment in the human genome such as chr18:14414. Thus, an alignment error may have occurred, and false-positive was reported.

### Two Variants on TUBB8 in Two Patients

**FIGURE 3:**
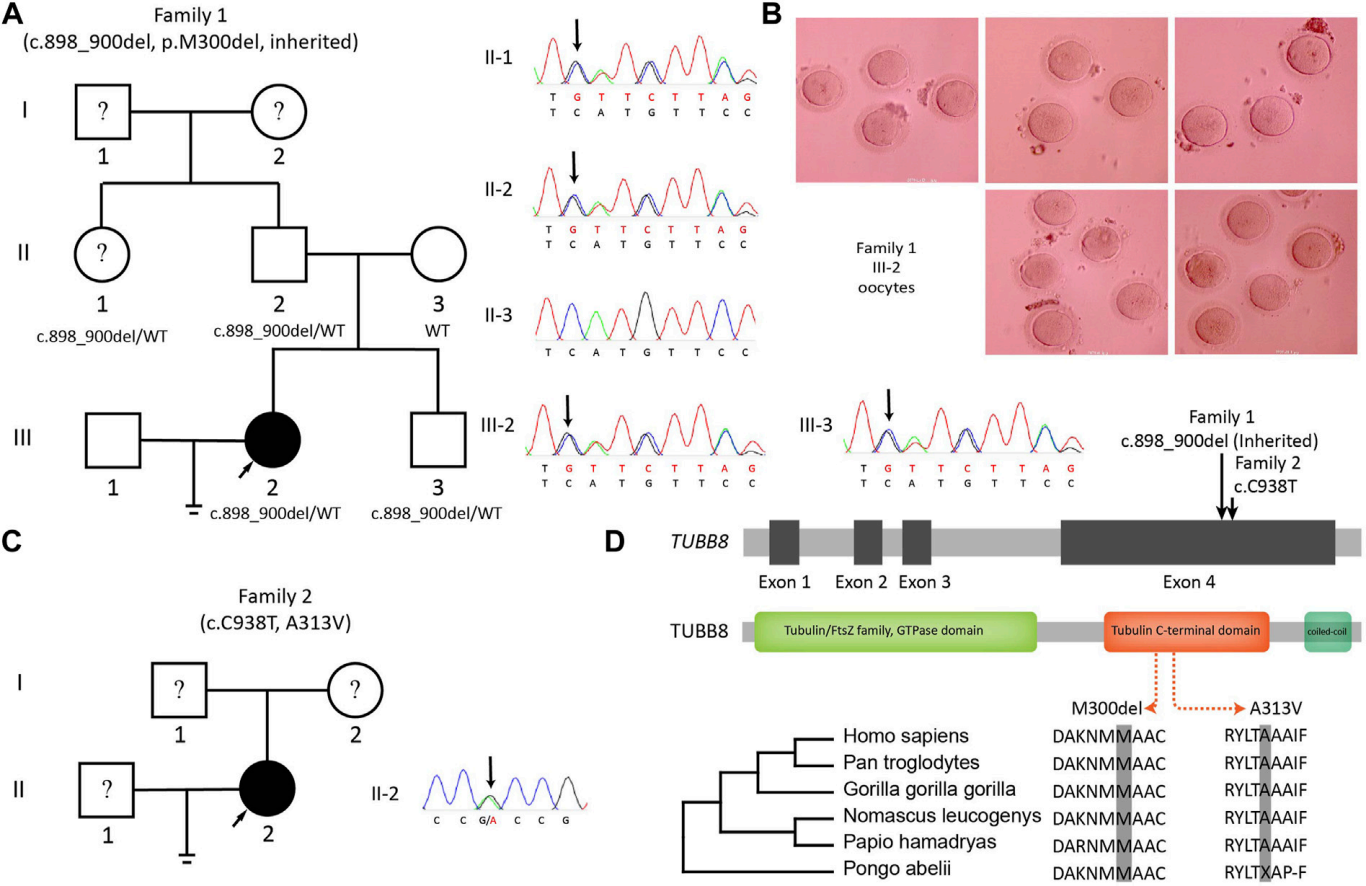
Variants on TUBB8 and its conservation. **(A)** The pedigree and Sanger sequencing of Family 1. **(B)** Oocytes from III-2 (Family 1) arrested in MI. **(C)** The pedigree and Sanger sequencing of Family 2. **(D)** Conservations of variant sites of TUBB8.

Two unrelated patients were found bearing variants on TUBB8. Patient III-2 in Family 1 was 30 years old and had been trying to conceive for 5 years. She had experienced two failed IVF cycles with 8 and 19 immature oocytes. One potentially significant variant on TUBB8 (c.898_900del:p.300_300del) was found. Sequencing of family members showed the paternal transmission of this heterozygous missense variant (Figure 3A). Oocytes typically arrested at MI stage are shown in Figure 3B. Patient II-2 in Family 2 was 29 years old and was diagnosed as RPL with two idiopathic spontaneous abortion (Figure 3C): a previously reported TUBB8 variant c.C938T:p.A313V with frequency of 0.007% in ExAC (Exome Aggregation Consortium, allele count: 8, allele number: 116668) and 0.05% in gnomAD (Genome Aggregation Database, allele count: 132, allele number: 273892). The position and conservation of the two SNVs are shown in Figure 3D.

### Disruption of Microtubule Network by Mutant TUBB8

**FIGURE 4:**
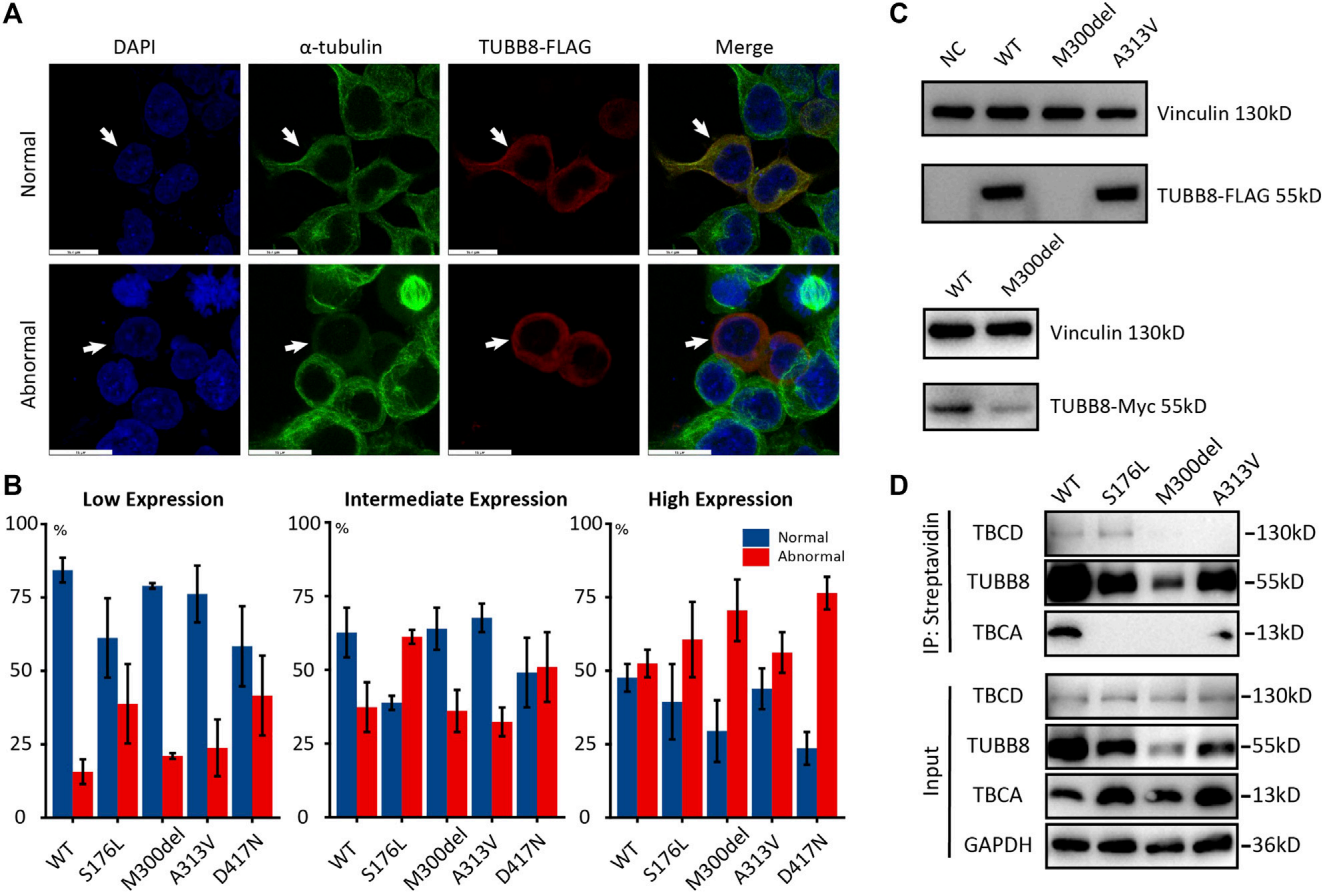
*In vitro* disruption of microtubule network by WT and mutant TUBB8. **(A)** Microtubule phenotypes resulting from expression of wild-type and mutated TUBB8-flag transfected in HEK293T. α-tubulin (green) showed the endogenous microtubule network, and M2-FLAG (red) detected the expression of the transgene. Microtubules assembled into a regular network in the normal group, while the abnormal group showed a mottled pattern. The bar in normal pattern indicates 15.3 µm, and the bar in the abnormal pattern indicates 15 µm. **(B)** The quantitative analysis of microtubule phenotypes in panel. Approximately 200 transfected cells were examined. I bar indicates standard error. **(C)** Western blot of overexpressions of wild-type and mutant TUBB8. NC was a blank load transfection negative control. WT was overexpressions of wild-type TUBB8 in HEK293T. M300del showed no visible protein band when detected with anti-flag antibody, while WT and A313V had comparable expression levels. When repeated with anti-Myc antibody, M300del still had a blurry band. **(D)** Co-IP of TUBB8 and tubulin cofactors. WT and A313V kept interacting with both TBCD and TBCA, and S176L lost binding with TBCA but maintained interaction with TBCD. M300del had no interaction with either TBCD or TBCA.

We next transfected WT and mutant TUBB8 vectors into cultured HEK293T cells to investigate the influence of variants on microtubule network. Two different morphologies were observed. On one hand, the normal cells had well-balanced α- and β-tubulin distributions which could assemble into a regular microtubule network. The endogenous α-tubulin (green) and overexpressed TUBB8 (red) showed a similar network co-assembling into a morphologically normal cell. On the other hand, abnormal cells lost regular microtubule network showing diffused distribution of α-tubulin and TUBB8 throughout the cytoplasm (Figure 4A). We observed the microtubule network with low, intermediate, and high transfection levels. Apart from the SNVs detected by our target sequencing M300del and A313V, we also compared two previously reported variants S176L and D417N. Under low expression, about 84.1% cells were morphologically normal in the WT group, and mutant groups were lower than WT: S176L was 59.87%, M300del was 78.61%, A313V was 77.13%, and D417N was 55.28%. With intermediate expressions, percentages of normal cells decreased, as WT dropped to 58.83%, and so did those of the mutant groups: S176L was 38.1%, M300del was 61.60%, A313V was 68.0%, and D417N was 49.08%. Under high expression levels, normal cells only took up 46.92% in the WT group, and S176L was 29.55%, M300del was 20.44%, while A313V was 41.99%, and D417N was 20.40% (Figure 4B and Supplementary Table S2). The different disrupted tendencies in these four mutant groups might indicate distinguished mechanisms. S176L, M300del, and A313V were predicted to impact dimer stability, β-tubulin folding, or polymerization, while D417N was predicted to disrupt microtubule network by influencing kinesin binding (Feng et al., 2016).

Western blot showed that M300del variant had an extremely decreased protein amount with no visible band, which could be a result of faster protein degradation by this variant. A313V had comparable expression levels with WT. We repeated western blot with a different tag and antibody, and M300del still showed a dramatic lowered expression, which implied the M300del variant resulted in a severely impaired protein function following a loss-of-function mechanism (Figure 4C).

### Interactions Between TUBB8 and Tubulin Co-Factors

**FIGURE 5:**
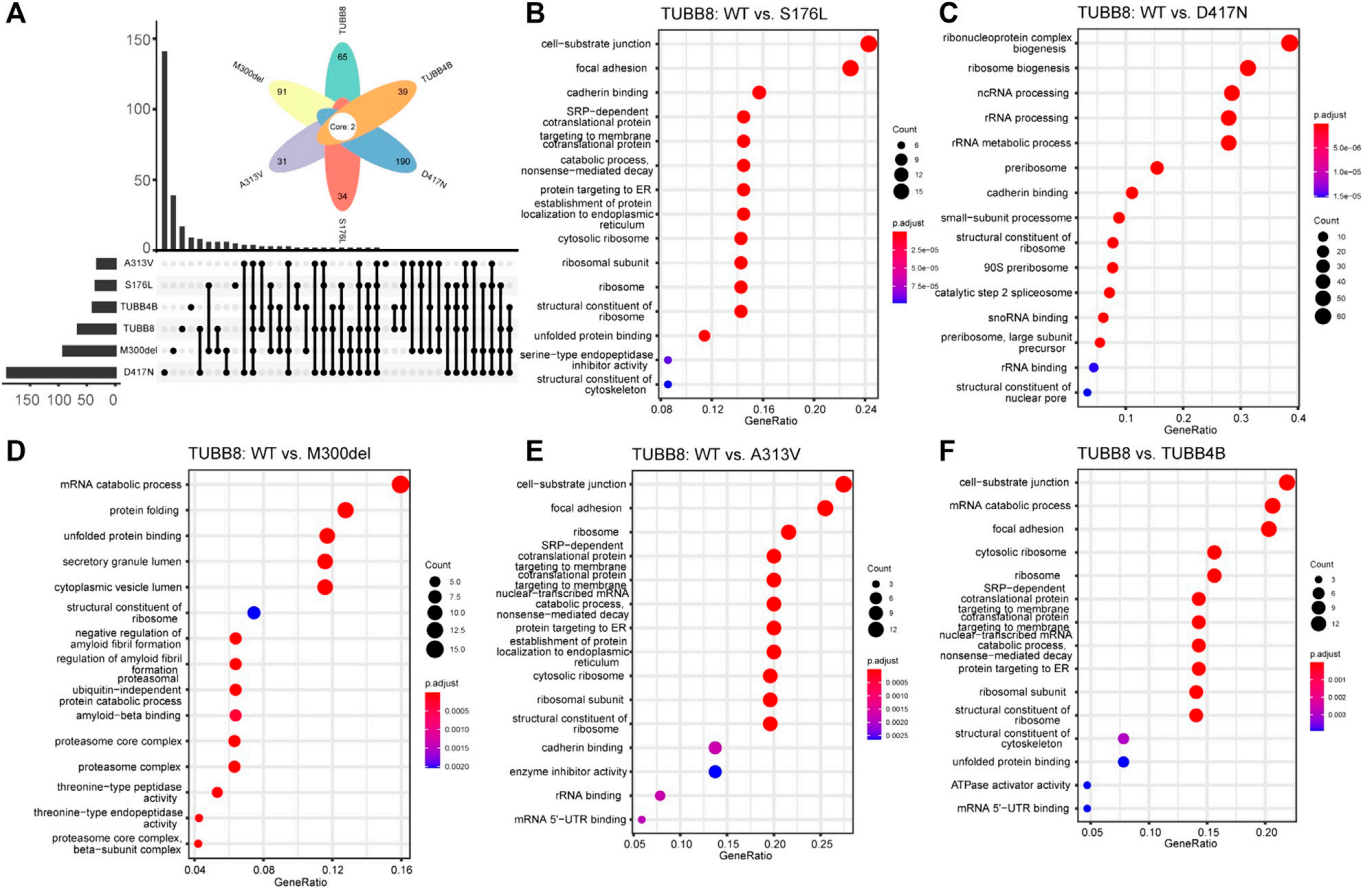
GO enrichment analysis of WT and mutant TUBB8. **(A)** Amounts of unique and common proteins between each group. **(B)** GO enrichment analysis of differently expressed proteins between TUBB8 WT and S176L. **(C)** GO enrichment analysis of differently expressed proteins between TUBB8 WT and D417N. **(D)** GO enrichment analysis of differently expressed proteins between TUBB8 WT and M300del. **(E)** GO enrichment analysis of differently expressed proteins between TUBB8 WT and A313V. **(F)** GO enrichment analysis of differently expressed proteins between TUBB8 and TUBB4B.

To identify the protein interactome of TUBB8, we transfected WT/mutant TUBB8 and TUBB4B vectors in HEK293T cells and purified proteins with M280 Dynabeads. Mass spectrometry was performed. Comparisons were made to investigate the specific interacting molecules of TUBB8 from a different β-tubulin isotype TUBB4B, so did WT vs mutant TUBB8 (S176L, M300del, A313V and D417N). Totally 1655 proteins were detected in seven groups including NC, which was a blank load transfection negative control. Differently expressed proteins were clustered using GO enrichment analysis. Figure 5A shows the uniquely expressed proteins of each group when compared with NC. The enrichment analysis of WT vs mutant TUBB8 is shown in Figures 5B–E. Comparing TUBB8 with TUBB4B, the main differently expressed proteins were enriched in the cell–substrate junction, mRNA catabolic process, and focal adhesion (Figure 5F). TUBB8 specifically expresses in human oocyte and early embryo while TUBB4B shows low tissue specificity. Therefore, this might lead to the different protein interactome, which is the basis of different protein functions. It is noted that M300del had been enriched to the ubiquitin-independent protein catabolic process (Figure 5D). Since the M300del variant showed a decreased protein expression in WB, we assumed that M300del activated a protein degradation pathway independent from ubiquitination which needs to be further investigated.

We further focused on how mutant TUBB8 disrupted microtubule network. According to previous studies, tubulin cofactors were vital in the process of tubulin heterodimer folding. During biogenesis and degradation of the α/β heterodimer, tubulin cofactors function synergistically on the folding of α- or β-tubulin monomers (Nithianantham et al., 2015). Co-immunoprecipitation showed that apart from S176L with increased expression of TBCD, other mutant TUBB8 did not change the expressions of TBCD or TBCA. M300del and A313V totally lost binding with TBCD, unlike WT or S176L. The absence of interaction between M300del/A313V and TBCD might destroy transport of the tubulin monomer and disrupt microtubule assembly. Interactions between TUBB8 and TBCA showed a different result. S176L and M300del had no interaction with TBCA, but WT/A313V still withheld the interplay (Figure 4D), assuming two varying mechanisms underlying the differently mutant TUBB8. TBCA mainly received β-tubulin from heterodimers and was critical in tubulin recycling (Nolasco et al., 2021). TBCD formed a trimer with regulatory GTPase ARL2 and β-tubulin (Francis et al., 2017). A previous report investigated S176L disrupting microtubule assembly by interfering GTP hydrolysis (Feng et al., 2016), so it could be speculated that the S176L variant was passed on by TBCD but failed to form heterodimers due to interfering GTP hydrolysis. M300del had no interaction with either TBCD or TBCA, implying its total failure in forming heterodimers, as M300del could not bind with tubulin cofactors at the first step. A313V could interplay with TBCA but not with TBCD. The possible reason was that M300del and A313V were buried within the protein structure, thus tubulin cofactors could not efficiently bind to M300del or A313V-bearing proteins, resulting in the destabilization of tubulin folding.

## Discussion

Despite the long-time use of genetic screening on other diseases, the practice of it on female infertility is still at the flagging stage. As pathogenic genes of female infertility have just been revealed in recent years, it is of great significance to develop an efficient and cost-effective screening method, especially for numerous idiopathic PI/RPL patients. To achieve this goal, we designed a target gene panel to sequence 22 known female infertility-related genes. Samples from 68 PI/RPL patients were sequenced with an average depth of 1559×. A sum of 3,134 exonic and splicing variants was detected, and potentially significant variants were further investigated. Based on this target sequencing, two TUBB8 variants were found including a novel one. We demonstrated mutant TUBB8 protein disrupting microtubule network and the loss-of-function role of M300del variant. We also revealed the interactome of WT and mutant TUBB8 and validated their different interaction patterns with TBCD and TBCA.

Panel sequencing enables specific in-depth detection of target genes. Its lower time-consumption, lower cost, and high accuracy make it a cost-effective tool for clinical application. Not only could our panel provide accurate diagnosis for patients but also could open a new window for the discovery of new pathogenic variants. For better performance, our panel needs optimization. First, the average depth of genes was unbalanced. FOXP3 had reached a mean depth of 2500×, while PATL2 did not achieve more than 1000×. The uneven depth of each gene might be a consequence of the innate characteristics of gene sequences. When genes like PATL2 have high abundance of GC or duplicated regions, capture efficiencies of specific probes might be affected. To improve the sequencing depth, extra or longer probes could be applied. Another concern is the false-positive reports in a particular position. When a target region was highly homologous with unwanted areas on the genome, it could be possible to enrich the unwanted regions or map the reads to the wrong place, thus leading to false-positive reports. One possible solution is longer probes or performing specific amplifications to increase the specificity of hybridization capture. Moreover, the current mean depth of our panel was 1559×, which might be excessive for genetic disorders (LaDuca et al., 2017). Usually 30× to 40× genome-sequencing would be adequate to give a confident sequence performance (Telenti et al., 2016), while the current clinical exome-sequencing uses 120× as a standard (Kong et al., 2018). The otoscope hearing loss panel achieved 716× per patient (Sloan-Heggen and Smith, 2016). Next, we will optimize our panel to have a balanced and reasonable sequencing depth for better performance and reduce the cost.

Some variants that we detected had no certain annotation in ACMG and InterVar. It might be that these variants are novel, or time was required for the database update. The newly published NyuWa Genome Resource providing the variation profile for Chinese population does not contain annotation of TUBB8:M300del or A313V. We also searched NHLBI Exome Sequencing Project, genomAD, human genetic variation database, ClinVar, and Online Mendelian Inheritance in Man (OMIM). We found that M300del had no annotation in these databases, and A313V was predicted to be likely pathogenic in ClinVar. The novel variant M300del expanded the spectrum of TUBB8 pathogenicity, and we confirm the pathogenic role of the two variants detected by our panel. To sum up, our target sequencing gene panel is unique in implementing to find novel and rare variants.

We also revealed that tubulin cofactors lost their binding to mutant TUBB8 in the process of heterodimer folding. TBCD passes on β-tubulin from TBCA and then transports them to the transient super-complex of TBCD/TBCE/TBCC trimer and α-tubulin (Nithianantham et al., 2015). However, M300del and A313V lost binding with TBCD, indicating the failed transport of β-tubulin monomer, which might lead to microtubule disruption. On the other hand, TBCA lost binding with S176L and M300del, indicating that mutants located in different domains of TUBB8 disturb different steps of heterodimer formation. A study on microtubule reconstruction and microtubule dynamics might help reveal further mechanisms. The interactome of M300del enriched in ubiquitin-independent protein catabolic process might imply that M300del proteins were eliminated through uncanonical pathways in an accelerated manner. For further study, more differently expressed proteins from mass spectrum would be validated, and downstream pathways and potential target molecules would be identified. It is promising to uncover deeper underlying mechanisms and bring new treatment strategies for variant-bearing patients. Finally, for the loss-of-function M300del, we are trying to conduct clinical trials of the WT TUBB8 supplement by microinjection of WT TUBB8 mRNA into the oocyte similar to previously reported (Sang et al., 2018).

The limitation of our work is the sample size. We are recruiting more patients and conducting optimization of the current sequencing panel. Moreover, we will add newly found female infertility pathogenic genes such as MEI1 (Dong et al., 2021) and FBXO43 (Weijie Wang et al., 2021). In conclusion, we designed a target-sequencing gene panel of female infertility-related genes and tested it in 68 patients. Two variants of TUBB8 were found, as well as a novel SNV M300del, and the primary loss-of-function mechanism of this novel variant was demonstrated. Further study will be aiming at both the optimization of this sequencing panel and the deeper understanding of TUBB8 pathology. We hope this target sequencing gene panel would be an efficient and cost-effective tool for genetic screen in reproductive centers to achieve a better diagnosis.

## Data Availability

The datasets presented in this study can be found in online repositories. The names of the repository/repositories and accession number(s) can be found below: https://ngdc.cncb.ac.cn/; [HRA002192].
