# Supplementary material for: Target-Sequencing of Female Infertility Pathogenic Gene Panel and a Novel TUBB8 Loss-of-Function Mutation
Source: Front Genet. 2022 May 10;13:865103. doi: 10.3389/fgene.2022.865103 (PMC9127544; doi:10.3389/fgene.2022.865103)
Supplement: Supplementary file 1 [file Table2.docx]

**Supplementary Table 2. Percentages of normal tubulin distribution of three independent immunofluorescence experiments showing WT/mutant TUBB8 in microtubule morphology.**

|  |  | WT | | |  | S176L | | |  | M300del | | |  | A313V | | |  | D417N | | |
| --- | --- | --- | --- | --- | --- | --- | --- | --- | --- | --- | --- | --- | --- | --- | --- | --- | --- | --- | --- | --- |
| Normal% |  | 1 | 2 | 3 |  | 1 | 2 | 3 |  | 1 | 2 | 3 |  | 1 | 2 | 3 |  | 1 | 2 | 3 |
| Low |  | 77.42 | 83.00 | 91.89 |  | 76.71 | 61.97 | 30.77 |  | 79.10 | 80.00 | 79.74 |  | 65.31 | 69.77 | 96.30 |  | 63.46 | 75.00 | 29.73 |
| Intermediate |  | 61.27 | 50.94 | 79.76 |  | 38.10 | 42.22 | 33.93 |  | 70.31 | 67.09 | 47.50 |  | 61.19 | 65.38 | 77.42 |  | 50.94 | 68.52 | 27.78 |
| High |  | 53.85 | 48.94 | 37.97 |  | 26.47 | 53.09 | 9.09 |  | 35.00 | 26.32 | 0.00 |  | 47.06 | 28.30 | 50.62 |  | 29.55 | 10.34 | 21.31 |
