# Supplementary material for: Target-Sequencing of Female Infertility Pathogenic Gene Panel and a Novel TUBB8 Loss-of-Function Mutation
Source: Front Genet. 2022 May 10;13:865103. doi: 10.3389/fgene.2022.865103 (PMC9127544; doi:10.3389/fgene.2022.865103)

Supplementary Figure 1. Sanger sequencing of some variants. a, b, c were three representative Sanger sequencing validations with FRE above 0.4. d, e were two unvalidated variants with FRE lower than 0.4.

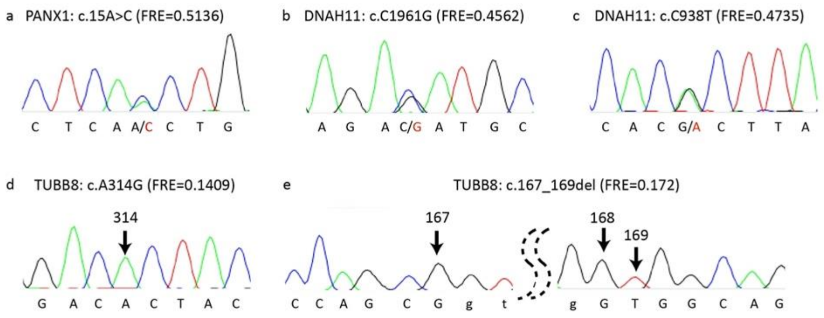

Supplement: Supplementary file 3 [file Image1.pdf]
